# Anti-inflammatory and Neuroprotective Agents in Clinical Trials for CNS Disease and Injury: Where Do We Go From Here?

**DOI:** 10.3389/fimmu.2020.02021

**Published:** 2020-09-10

**Authors:** Khalil Mallah, Christine Couch, Davis M. Borucki, Amer Toutonji, Mohammed Alshareef, Stephen Tomlinson

**Affiliations:** ^1^Department of Microbiology and Immunology, Medical University of South Carolina, Charleston, SC, United States; ^2^Department of Health Sciences and Research, College of Health Professions, Medical University of South Carolina, Charleston, SC, United States; ^3^Department of Neurosciences, Medical University of South Carolina, Charleston, SC, United States; ^4^Medical Scientist Training Program, Medical University of South Carolina, Charleston, SC, United States; ^5^Department of Neurological Surgery, Medical University of South Carolina, Charleston, SC, United States; ^6^Ralph Johnson VA Medical Center, Charleston, SC, United States

**Keywords:** clinical trials, stroke, traumatic brain injury, complement inhibition, multiple sclerosis, amyotrophic lateral sclerosis, neuromyelitis optica, Parkinson's disease

## Abstract

Neurological disorders are major contributors to death and disability worldwide. The pathology of injuries and disease processes includes a cascade of events that often involve molecular and cellular components of the immune system and their interaction with cells and structures within the central nervous system. Because of this, there has been great interest in developing neuroprotective therapeutic approaches that target neuroinflammatory pathways. Several neuroprotective anti-inflammatory agents have been investigated in clinical trials for a variety of neurological diseases and injuries, but to date the results from the great majority of these trials has been disappointing. There nevertheless remains great interest in the development of neuroprotective strategies in this arena. With this in mind, the complement system is being increasingly discussed as an attractive therapeutic target for treating brain injury and neurodegenerative conditions, due to emerging data supporting a pivotal role for complement in promoting multiple downstream activities that promote neuroinflammation and degeneration. As we move forward in testing additional neuroprotective and immune-modulating agents, we believe it will be useful to review past trials and discuss potential factors that may have contributed to failure, which will assist with future agent selection and trial design, including for complement inhibitors. In this context, we also discuss inhibition of the complement system as a potential neuroprotective strategy for neuropathologies of the central nervous system.

## Introduction

Brain and neural injury is a non-specific disease category that includes traumatic brain injury (TBI), stroke, and intrinsic neurodegenerative diseases. Combined, these disease processes affect over 4 million people in the U.S. annually (1–6). A challenge in designing medical treatments for neurodegenerative diseases is the location and multifactorial nature of the pathologies, which are mostly complex and involve dysfunction of multiple homeostatic processes. Common links between these pathologies include metabolic disruption, cellular degeneration, protein aggregation, alterations in neurotransmitter signaling, and an ongoing neuroinflammatory response (7, 8). Anti-inflammatory and immune-modulating agents are gaining an increased level of interest for treating neurodegenerative diseases and conditions (9, 10). The goal of treatment has shifted from symptomatic management to approaches for neuroprotection and regeneration (11–15). Nevertheless, therapeutic success of various agents in pre-clinical models has largely failed to translate to success in clinical trials (16–18). With this in mind, the complement system is being increasingly discussed as an attractive therapeutic target for treating brain injury and neurodegenerative conditions, due to emerging data supporting a pivotal role for complement in promoting multiple downstream activities that promote neuroinflammation and degeneration.

The complement system is a collection of plasma and membrane proteins that together function to promote and modulate both innate and adaptive immune responses (19). There are three main pathways of complement activation: the classical, lectin, and alternative pathway (20). The classical pathway is mainly activated following the binding of C1q to Fc domains of antibodies. The lectin pathway is initiated by the binding of sugar recognition molecules (mannose binding lectin, ficolins, and collectins) to specific carbohydrates. The alternative pathway can be activated by spontaneous cleavage of C3, but is also an amplification loop for the other pathways. All three activation pathways converge at C3 cleavage, which produces C3a and C3b by an assembled C3 convertase. C3a is a soluble peptide, while C3b becomes bound to the activating surface and is further cleaved to iC3b and C3d, which function as opsonins recognized by immune cell receptors. Further downstream, n assembled C5 convertase cleaves C5 to produce C5a and C5b. Both the C5a and C3a products (anaphylatoxins) have pivotal roles in regulating innate and adaptive immune response (21). The C5b product initiates assembly of the terminal cytosolic membrane attack complex (MAC or C5b-9). Aberrant and over activation of the complement system is closely associated with inflammatory responses seen in multiple diseases and disease conditions, including neuroinflammatory responses in central nervous system (CNS) pathologies (22). For example, complement activation after TBI results in ongoing microglia and astrocyte activation, a reduction in dendritic and synaptic density, and inhibition of neuroblast migration (23). It has also been shown that following stroke in a murine model, microglia perform inappropriate synaptic pruning via a complement-dependent mechanism involving C3 opsonins (24). Similar complement-mediated processes are indicated in other CNS diseases, such as Alzheimer's disease (25) and Multiple Sclerosis (MS) (26). The C3a and C5a anaphylatoxins are also implicated in a variety of CNS disease pathologies via their chemotactic and cell activation properties resulting from their interaction with receptors on immune cells (27). Cell activation by complement activations products, including the terminal MAC, can result in the generation of multiple inflammatory molecules, such as cytokines, chemokines, and reactive oxygen species (28–30). Considering the multitude of anti-complement therapeutics now in clinical development (31), and with regard to preclinical data and trial design for the testing of new neuroprotectants, including complement inhibitors, we believe it will be informative to review anti-inflammatory and neuroprotective agents that have been investigated in clinical trials, even though most ended in failure. In Supplementary Table 1, we highlight the different drug classes and most of the clinical trials discussed in this review.

## Stroke

**Figure 1:**
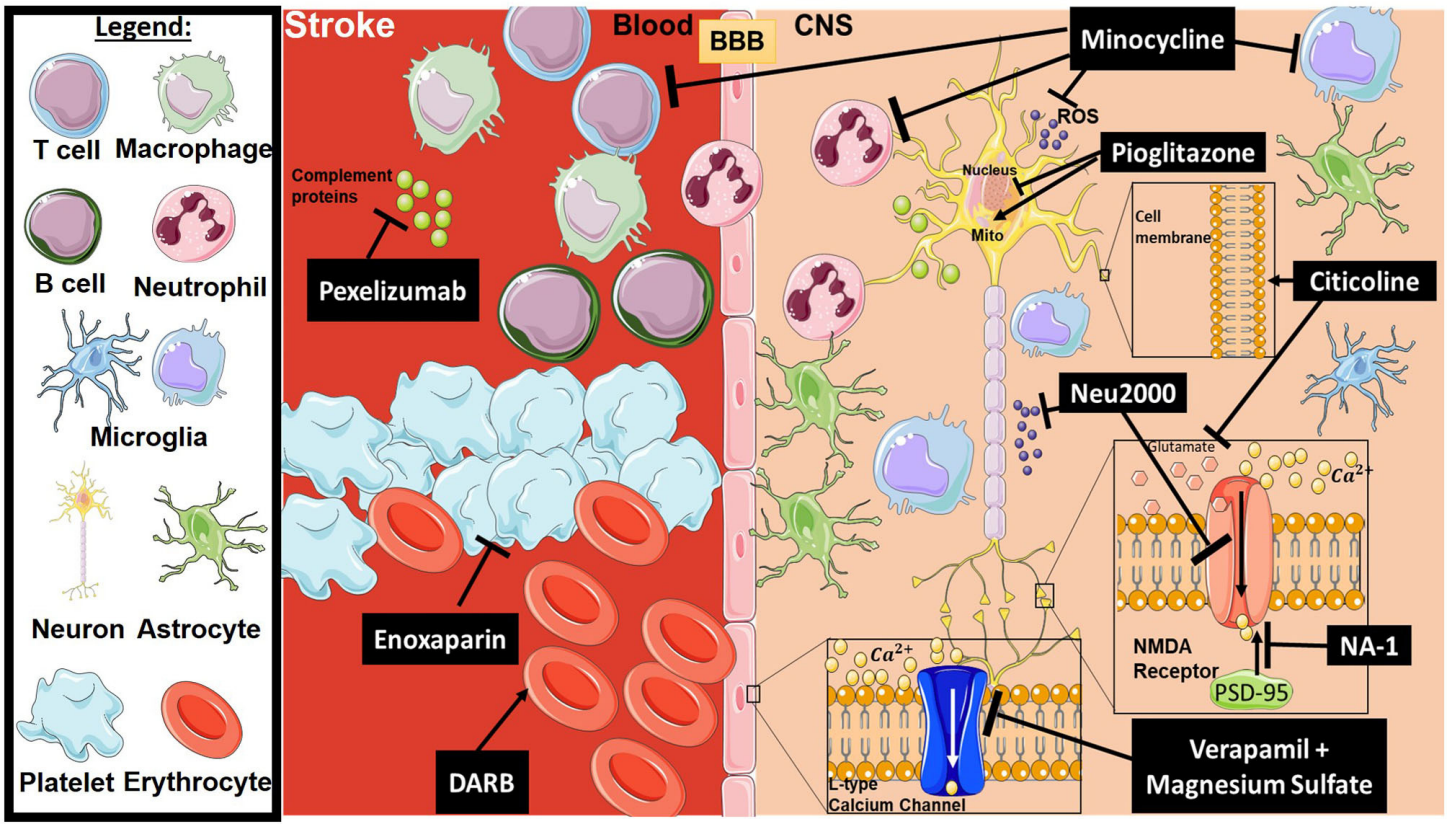
Mechanism of action for the therapeutics discussed in stroke injury section. Lines ending with a flat dash indicate an inhibitory effect, and lines ending with arrows indicate a positive/stimulating effect. In brief, pexelizumab is a monoclonal antibody derivative which blocks complement at C5 activation. Minocycline is an immune-modulator which exhibits several inhibitory actions on activated microglia, neutrophils, T-cells, ROS, and others. Pioglitazone stimulates mitochondrial biogenesis and inhibits transcription of genes implicated in fatty acid oxidation. Citicoline stimulates stabilization of the cellular membrane and prevents excessive release in glutamate along with other functions. Neu2000 is a subtype-selective inhibitor of NMDA receptor and a ROS scavenger. NA-1 disrupts the interaction between PSD-95 and NMDA receptors, thus preventing overactivation of the NMDA receptor resulting in excitotoxicity. Verapamil and Magnesium sulfate block overactivation of L-type calcium channels, which will inhibit intracellular calcium dysregulation. Enoxaparin is an anti-coagulant, and DARB is a erythropoiesis-stimulating agent. All drawings of cells/molecules used in this figure were obtained and modified from Servier Medical Art by Servier, licensed under a Creative Commons Attribution 3.0 Unported License (https://smart.servier.com/).

Stroke is the second leading cause of death disability worldwide (32). Current therapies for acute ischemic stroke are reperfusion-based and consist of endovascular thrombectomy (EVT) and tissue plasminogen activator (tPA), the latter being the only approved pharmacological treatment for acute ischemic stroke (33, 34). However, in large part because of the short treatment window (4.5 h) and a requirement for neuroimaging to rule out intracerebral hemorrhage, tPA is used in only up to about 7% of patients suffering from acute ischemic stroke in the United States (33). While the effectiveness of EVT has been confirmed with a treatment window up to 24 h after symptom onset, there are limitations to its widespread use. The procedure can only be used in ischemic stroke patients with a proximal large artery occlusion, and many hospitals and stroke centers do not have the capability to perform the procedure. In addition, reperfusion therapy does not appear to prevent a subsequent neuroinflammatory response after stroke, and while successful reperfusion can improve motor function outcome, it does not appear to prevent cognitive decline (35). Thus, there remains an interest in developing neuroprotective strategies to treat stroke, even though very few neuroprotective agents have been shown to be unequivocally beneficial in randomized controlled clinical trials (36, 37). Figure 1 provides a representative scheme of the mechanism of action for the discussed therapeutics in stroke.

### Complement Inhibitors

Pexelizumab is a single-chain antibody fragment derived from eculizumab, a Food and Drug Administration (FDA) approved anti-C5 monoclonal antibody that blocks C5 cleavage and the generation of C5a and the MAC (38). Stroke is a serious complication of coronary artery bypass graft (CABG) surgery due to an undesirable systemic inflammatory response (39). With this in mind, a 2003 phase II clinical trial assessed the use of Pexelizumab as a potential treatment in CABG patients to reduce complement-mediated tissue damage and associated systemic inflammation, with the goal of reducing neurological injury and deficits (40). Primary endpoints were not met, and the study showed no significant improvement in cognition or neurological deficits. There was, however, an improvement in visuospatial function, although this study was not followed up. Due to the fact this trial was purely observational, stroke was primarily assessed as an association of the number of strokes in hospital and 3-month patient outcomes, as opposed to a cause and effect relationship. Additionally, stroke was not the primary disease analyzed in this trial and was a retrospective study, filtered as a secondary incidence. Another phase III clinical trial was planned to assess the efficacy and safety profile of Pexelizumab, although no further data or information was recorded following this trial (Clinical Trial Identifier: NCT00048308).

### Thiazolidones (Pioglitazone)

Pioglitazone is commonly used as an oral drug to reduce insulin resistance in Type II Diabetes. It is a peroxisome proliferator-activated receptor gamma (PPARγ) agonist and alters the transcription of genes implicated in fatty acid oxidation, which result in metabolic changes (41). A variety of neuroprotective mechanisms have been attributed to PPARγ, including the induction of genes involved in oxidative stress defense, induction of anti-inflammatory responses, and stimulation of mitochondrial biogenesis (42). Pioglitazone was shown to provide effective neuroprotection in an animal model of ischemic stroke, although the exact mechanism of action of its neuroprotective effect was not determined (43). Pioglitazone was evaluated in a clinical trial in acute stroke patients with hyperglycemia, but due to difficulty with participant enrollment, the phase II trial was terminated in 2016 (NCT02195791). The most recent phase III trial (NCT00091949), completed in 2015, analyzed the efficacy of Pioglitazone in preventing future strokes in non-diabetic patients who had previously suffered from an ischemic stroke. One major limitation of this clinical trial that was noted is that all participants had insulin resistance, with data analysis focusing on the prediabetic group. Therefore, with diabetes being the true end point in this trial, the true efficacy of Pioglitazone in stroke patients who are non-diabetic was not discernable.

### Immune-Modulator

Minocycline is a second-generation, semi-synthetic tetracycline effective against gram-positive and gram-negative bacteria, and has been in therapeutic use for over 30 years (44). It has also been reported that Minocycline can exert anti-inflammatory and neuroprotective properties due to its inhibitory effect on microglial activation. A small number of preclinical studies demonstrated that Minocycline improved post-stroke outcomes, which led to it being evaluated in a Phase I and II trial in stroke patients in 2012. Minocycline was shown to be well-tolerated alone and in combination with tPA (45) (NCT00630396). However, no conclusion could be drawn with regard to the optimal tolerated dose due to insufficient recruitment of participants in the lower dose groups. In a subsequent pilot study completed in 2016 (NCT01805895), Minocycline's anti-inflammatory effects and pharmacokinetics in acute cerebral hemorrhage participants was investigated. Although the half-life of minocycline was compatible to the previous Phase I trial, there was a delayed oral absorption in this gravely ill patient group. The results from this trial found no significant differences between inflammatory profiles in the placebo vs. treated patient groups, but limitations of this trial included small sample size, a shortened enrollment window of at most 24 h, and participant heterogeneity. Future studies to assess efficacy and anti-inflammatory properties would benefit from enrolling larger and more severe stroke patient populations, as well as comparing intravenous vs. oral administration at a more chronic timepoint. Minocycline continues to be investigated in trials for stroke and neuroinflammation.

### Calcium Channel Blockers

Verapamil is an L-type calcium channel blocker used to treat angina and hypertension, and is also used to treat cerebral vasospasm secondary to subarachnoid hemorrhage (46). It was the first calcium channel antagonist to be introduced into therapy (in the early 1960s) (47). As anti-hypertensive drugs, calcium channel blockers target a modifiable risk factor for stroke, but additional evidence indicate they have a neuroprotective action based on their ability to minimize carotid intima-media thickening (48) and the fact that intracellular calcium dysregulation can trigger ischemic cell death (49). A phase I clinical trial, completed in 2016, demonstrated neuroprotective activity of Verapamil when administered together with tPA and/or mechanical thrombectomy (NCT02235558). Combining the use of Verapamil with direct intra-arterial administration rapidly restored the cerebral artery, and may offer an opportunity to translate bench-side neuroprotective effects into bed-side success (46). This SAVER phase 1 clinical trial revealed the addition of Verapamil to thrombectomy, produced no thromboembolic complications. Due to the limited sample size of only 11 patients, this study did not have the power to demonstrate Verapamil's efficacy. Future studies should assess neuroprotection in regard to comorbidities and gender. There is an ongoing phase I study testing this agent for neuroprotection in stroke patients, which is estimated to be completed in the spring of 2020 (NCT03347786).

Magnesium sulfate has been investigated as a calcium channel blocker, as well as an anticonvulsant, a cardiovascular drug, an anesthetic, a tocolytic agent, an anti-arrhythmia drug, and an analgesic (PubChem CID: 24083). Magnesium sulfate has shown neuroprotective effect post-stroke in several animal models (50–53). However, a randomized clinical trial in stroke patients showed no overall benefit when magnesium sulfate was administered at times of ~7.5 h after stroke symptom onset (NCT01502761). An additional exploratory analysis was performed, and indicated a suggested, but non-significant, potential efficacy in a subgroup of patients with intracerebral hemorrhage treated within the first 3 h after symptom onset (54). A phase III trial completed in 2015 analyzed magnesium sulfate therapy when administered within 2 h after stroke, but although the therapy was safe, overall patient disability outcomes were not improved at 90 days post-stroke (55) (NCT00059332). Future studies could benefit from the inclusion and analysis of comorbidities, such as hypertension, hyperlipidemia, diabetes and visual impairment, as well as hospital readmission rates to more accurately reflect stroke care and stroke patient outcomes.

### Multi-Target Neuroprotectants

Neu2000 was designed as a multi-target neuroprotectant that combines both NR2B subtype-selective blockade of the N-methyl-D-aspartate (NMDA) receptor and reactive oxygen species (ROS) scavenging (56), which are both connected to brain cell death in stroke. The therapeutic potential of Neu2000 has been shown in several animal models of stroke, and it has better efficacy and longer therapeutic window than either NMDA receptor antagonist or anti-oxidants alone (57). A phase II clinical trial completed in 2018 evaluated Neu2000 as an adjunct neuroprotective agent together with EVT in patients presented to stroke centers within 8 h of acute ischemic stroke (58) (NCT02831088). There has been no further update on the status of this trial.

Citicoline, or cytidine 5'-diphosphocholine (CDP-choline), a drug that combines neurovascular protection and repair promoting effects, has been used to treat acute ischemic stroke and other neurological disorders, and it has an excellent safety profile (59). Citicoline is a water-soluble compound, and pharmacokinetic studies on healthy adults have shown good absorption with both oral and intravenous routes of administration (60). Once absorbed, Citicoline is converted to choline and cytidine, which enter the systemic circulation and crosses the blood-brain barrier, where it is resynthesized into citicoline in the brain. Citicoline has been shown to possess several protective functions including promoting membrane stability, and inhibiting glutamate excitotoxicity, apoptosis, and oxidative stress (61, 62). In an experimental stroke model, citicoline increased SIRT1 (Citicoline-like activator) protein levels in the brain concomitant with neuroprotection (63). Doses of up to 2,000 mg have been administered in multiple clinical trials. A 2016 meta-analysis of acute ischemic stroke patients who received the highest dose of Citicoline in the first 24 h without tPA treatment showed improvements (64). Citicoline has a long therapeutic window compared to tPA, although as an adjunct to tPA, it offered limited benefit. In a hospital based study published in 2019, the efficacy of Minocycline was compared to placebo in acute ischemic stroke patients (65). There was no statistical significance between both placebo and treatment groups, when assessing improved functional outcomes at discharge and 90 day follow up. Future studies should optimally incorporate not only assessing stroke functional recovery using the NIHSS, Modified Rankin Score, or Barthel Index, but also assess cognitive recovery, per the Mini Mental State Examination, which would better represent stroke outcomes in their entirety.

### NA-1

Nerinetide, more commonly known as NA-1, is a 20 amino acid peptide with the last 9 residues joined to the protein domain of human immunodeficiency virus type 1 (HIV-1) Tat protein, which confers cell permeable properties to the drug (66–68). Its mechanism of action, as determined in animal models of stroke, is to disrupt the interaction of the scaffolding protein, PSD-95, with NMDA receptor, thus preventing receptor signaling and protecting neurons from excitotoxicity (67–70). The ESCAPE-NA1 trial is the first phase III randomized controlled trial of neuroprotective medication in stroke patients in the context of endovascular thrombectomy. In this trial completed in 2019, the primary endpoint was a reduction in disability score in acute stroke patients with a small infarct core identified for abrupt EVT (71) (NCT02930018). Nerinetide did not show benefit in a majority of patients who had successful clinical outcomes after EVT when compared with those receiving placebo. However, although the primary outcome measure was not met, when patient groups were split based on t-PA treatment or not, the group that did not receive t-PA had significantly better outcomes and smaller infarct volume with NA-1 treatment. The main outcome assessed was functional recovery by the Modified Rankin Scale, which is not optimal for a neuroprotective clinical trial when the therapeutic agent is an adjunct to reperfusion techniques. This is because of the large size effect of reperfusion alone and the absence of a non-reperfused patient group. Limitations of the study included the fact that the trial was structured for stroke participants who were selected for thrombectomy. Secondly, no cognitive tests were performed during this trial or at least were not published. An alternative trial design would be to run two parallel trials comparing Nerinetide with placebo, one with and one without alteplase.

### Erythropoiesis-Stimulating Agents

Darbepoetin alfa (DARB) is in a class of medications called erythropoiesis-stimulating agents (ESAs) that stimulate the bone marrow to produce red blood cells. It is also used to treat anemia in patients afflicted with kidney failure, as well as anemia caused by chemotherapy in patients with certain types of cancer. It is not uncommon for patients who undergo high-risk surgical procedures to develop ischemic CNS complications. In theory, administering a neuroprotective agent before such a surgery would improve the outcome of any subsequent ischemic CNS injury. In this context, a prospective adaptive dose-finding trial of prophylactic DARB was initiated in a phase II clinical trial in 2008, but the trial was terminated prematurely following the publication of an erythropoietin stroke study showing possible harm (NCT00647998). Enrollment was halted before dose adjustments. Prior to surgery, nine patients received 1 mg/kg IV DARB, and there were no significant effects of prophylactic DARB on clinical outcome or CSF markers of neurologic injury in this pilot study, although all point estimates favored treatment (72). Subsequent clinical trials should identify the optimal dose of DARB, as well as determining safety and efficacy in larger stroke patient sample sizes.

### Anticoagulants

Enoxaparin, a low molecular weight heparin, is an anti-coagulant medication that is given as an injection. It is FDA approved and used to treat pulmonary embolism or deep vein thrombosis. Of relevance, it has been shown that anti-coagulant heparin can inhibit leukocyte accumulation in ischemic tissue by several mechanisms, including inhibition of adhesion molecule function and heparinase activity (73). Completed in 2018, the TEACH pilot randomized clinical trial compared antithrombotic drugs in hematological cancer patients who were afflicted with acute ischemic stroke. There was a failure in patient enrollment due to patient aversion to receiving injections. In addition, 40% of patients who were randomized to receive Enoxaparin ended up using aspirin due to discomfort with receiving injections (74) (NCT01763606). There is thus a lack of data to currently see a clear path forward for this drug, but future studies would need to assess the safety and feasibility of this therapeutic approach in this high-risk patient population in Phase 1 or 2 clinical trials. Additional studies could also compare aspirin to other oral antithrombotics which are FDA approved, such as Dabigatran, Rivaroxaban, or Apixaban, as opposed to injectables. Considering the failure in patient enrollment due to discomfort of injection, a focus on oral administration of antithrombotic reagents would be warranted.

## Traumatic Brain Injury

**Figure 2:**
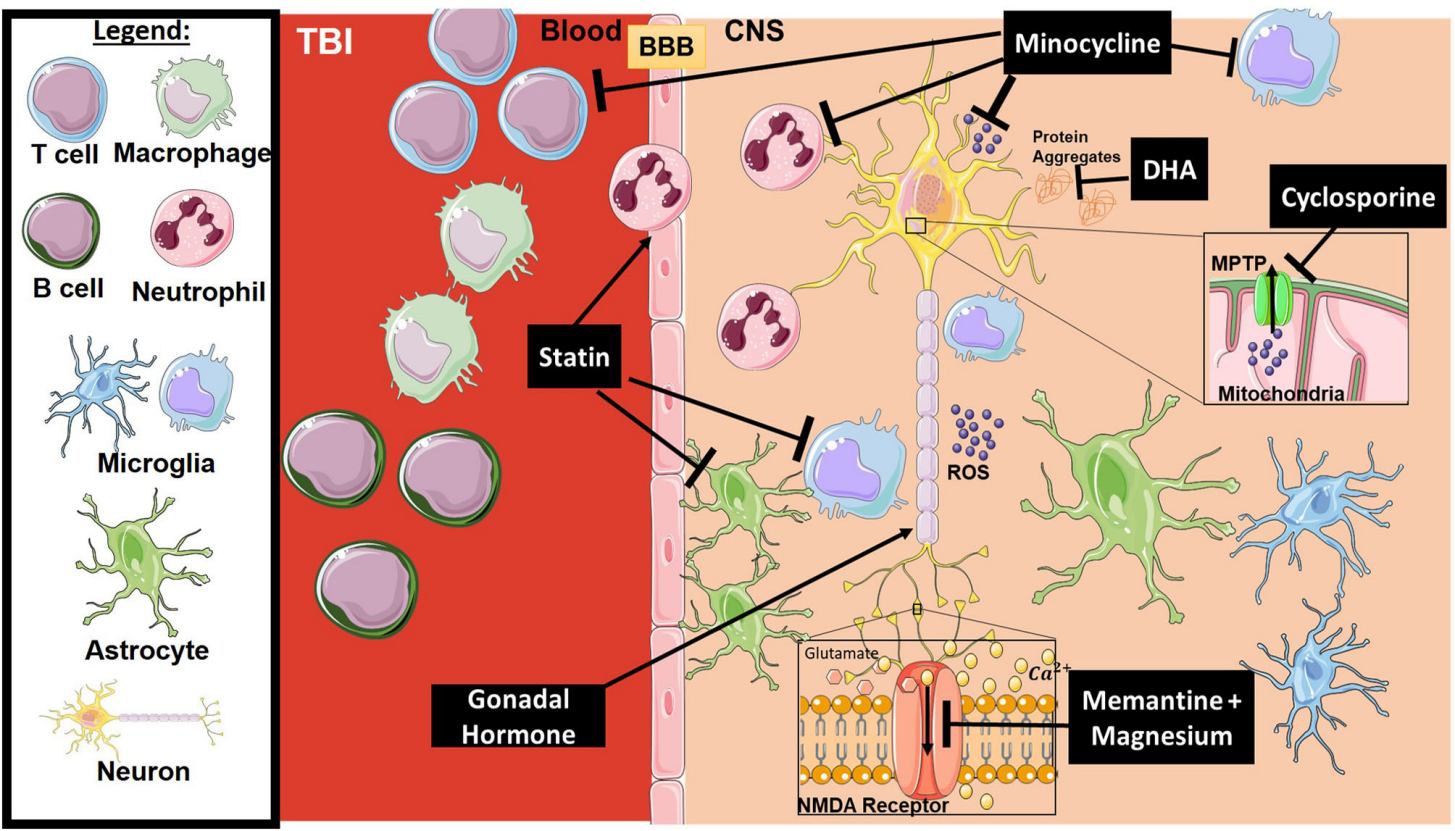
Mechanism of action for the therapeutics discussed in TBI section. Lines ending with a flat dash indicate an inhibitory effect, and lines ending with an arrows indicate a positive/stimulating effect. In brief, Minocycline is an immune-modulator which exhibits several inhibitory actions on activated microglia, neutrophils, T-cells, ROS, and others. Cyclosporine is an immune-modulator that inhibits the MPTP, thus inhibiting caspase cascade activation and ROS release. DHA is responsible for preventing protein accumulation (such as amyloid precursor protein APP), along with other functions. Statins, such as atorvastatin, play a stimulatory role on enforcing blood-brain barrier integrity and inhibition of glial activation. Gonadal hormones promote neuronal survival. Memantine and Magnesium inhibit overactivation of the NMDA receptor resulting in dysregulation of intracellular calcium and receptor toxicity. All drawings of cells/molecules used in this figure were obtained and modified from Servier Medical Art by Servier, licensed under a Creative Commons Attribution 3.0 Unported License (https://smart.servier.com/).

Traumatic brain injury is a form of acquired brain injury resulting from an external blow or jolt to the head and leading to an altered mental state. The injury itself is divided into two main phases: primary and secondary, which lead to temporary or permanent neurological changes (75). The primary phase is the direct mechanical damage to the brain, which is non-predictable and non-preventable. The secondary phase is a combination of physiological responses to the primary insult, such as excitotoxicity and an ongoing inflammatory process that includes immune system access to the mechanically injured tissue (76, 77). Current treatments for TBI patients include anti-anxiety, anti-coagulant, and anti-depressant drugs, but there is no approved neuroprotective agent for protection against a secondary phase of inflammation and injury that can persist for months to years after the initial insult. Below, we review several neuroprotective agents that have been investigated in clinical trials, although most have failed to show any benefit. Figure 2 provides a representative scheme of the mechanism of action for the discussed therapeutics in TBI.

### NMDA Receptor Modulators

Memantine is an uncompetitive low-affinity NMDA receptor antagonist with neuroprotective effects that is used to treat dementia in Alzheimer's patients. Located at synaptic sites, NMDA receptors are essential for controlling calcium influx into neurons and activity-dependent re-establishment of synaptic strength (78, 79). Overactivation of NMDA receptors results in increased calcium influx into the cell, leading to membrane depolarization, production of ROS species, cellular toxicity, and neuronal death (80). This mechanism of NMDA-mediated damage has been shown to occur in several CNS injury models, including TBI and stroke (81). In animal models of TBI, memantine treatment decreased the accumulation of phosphorylated tau proteins in the cortical tissue at early, although not chronic time points (up to 30 days post-injury), and suppressed microglial activation (82). A similar study showed that memantine decreased cerebral infarct area, increased neuronal survival in the perilesional hemisphere, and decreased levels of microgliosis and astrogliosis (83). Unfortunately, success in preclinical TBI studies has not been mirrored in clinical studies (84), with a principle reason being failure in patient recruitment. In the U.S., there have been two trails carried out in the past 15 years. The first study (NCT00462228) was a phase IV clinical trial which was terminated in 2013 due to lack of recruitment. The second trial (NCT02240589) was completed in 2017, but results were inconclusive with the main limitation being small sample size (11 participants).

Magnesium has multiple roles in cellular function and nerve transmission, and interacts with and blocks the calcium channel within NMDA receptors in a regulatory manner (85), preventing overactivation of NMDA receptors and glutamatergic excitatory action. In a study evaluating patients 6 months after suffering severe TBI (GCS 3–12 upon administration), 81% percent of patients that showed a poor neurological outcome had a significant decrease in serum magnesium levels (86). Of three clinical trials registered in clinicaltrials.gov to study the efficacy of magnesium sulfate for the treatment in TBI, only one was completed (87) (NCT00004730). The study concluded that infusion of magnesium for the first 5 days after injury was not neuroprotective. Furthermore, patients receiving higher doses of magnesium had increased mortality. Although recruitment was not an issue for this study (499 participants), inclusion criteria based on GCS was very broad, which included participants that fell in the range of 3–12 on the GCS scale, and even those that were intubated. The authors did subsequently subdivide the groups into severe intubated and severe non-intubated (GCS 3-8), but the latter group would still have high variability in injury outcomes. Further dividing patients into more narrow subgroups based on similarity in GCS scores may be a consideration for further studies.

### Immune-Modulator

Minocycline is a tetracycline antibiotic that possesses anti-inflammatory activity via modulation of enzyme activities, inhibition of apoptosis, inhibition of immune cell activation, and inhibition of cell proliferation (44). In mouse models, minocycline, administered either before or after TBI decreased lesion size and improved behavioral outcome acutely (88). Minocycline administration at early time points was also linked to a reduction in microglial activation and interleukin-1β expression, but not neutrophil infiltration (89). Furthermore, at a chronic time point of analysis (45 days) after acute minocycline treatment, levels of several inflammatory markers in serum and tissues were reduced to levels seen in non-injured sham controls (90).

A clinical trial to test the safety and feasibility of minocycline in acute TBI found that treatment had no impact on serum levels of S100 calcium binding protein B (S100-B), a well-established TBI biomarker, and no infections were recorded in liver function tests up to 12 months post-treatment, thus concluding that minocycline is safe for patients suffering from TBI (91) (NCT01058395). In another clinical study, minocycline was administered over a 12-week period to patients who had been subjected to a moderate-to-severe TBI at least 6 months prior. Patients were then reassessed 6 months after the beginning of treatment (92). Minocycline reduced the level of microglial activation but increased neurodegeneration as measured by axonal protein neurofilament light (NFL) in plasma. Thus, minocycline treatment was not beneficial, and the findings hint that that microglial activation in the chronic phase post-injury benefits the recovery of damaged axons. One limitation of this study was the very low patient numbers, which according to the authors was the reason they did not report clinical measures of drug effect. Another limitation was the focus on only microglia as a key cell type to determine efficacy of minocycline. Other cell types implicated in TBI such as astrocytes have been demonstrated to have an altered phenotype after minocycline treatment in pre-clinical models of TBI (93). Finally, the study relied on only one plasma marker, NFL, to assess neurodegeneration and efficacy of treatment.

Cyclosporine is an immune-modulator mainly used to treat transplant recipients. However, it also has neuroprotective properties via inhibiting the opening of the mitochondrial permeability transition pore (MPTP), which otherwise results in oxidative phosphorylation and rupture of the mitochondrial outer membrane, leading to the caspase cascade activation and neurodegeneration (94, 95). After murine TBI, cyclosporine resulted in a significant decrease in the production of ROS, key players in the ongoing excitotoxic effect after TBI, and restored mitochondrial membrane potential (96). A phase II study investigating the pharmacokinetics and safety of cyclosporine (NeuroSTAT) in patients with severe TBI [patients with a Glasgow Coma Scale (GCS) 4–8 were included] demonstrated it was safe and well-tolerated, with a trend in decreased cerebrospinal fluid levels of several TBI biomarkers, including glial fibrillary acidic protein (GFAP, an astroglial injury marker), NFL, Tau, and ubiquitin carboxyl-terminal hydrolase isozyme L1 (UCHL-1, a neuronal cell body injury marker) (97) (NCT01825044). Of note, only 16 patients were enrolled in this open-label trial, and a randomized trial with a larger number of participants would be required to draw any conclusions on the efficacy of cyclosporine for treating TBI. Nevertheless, as with minocycline, cyclosporine is an immune-modulator that may not be an optimal therapeutic approach for patients with TBI at increased risk of infection.

### Statin Treatment (Atorvastatin)

Statins are a class of lipid-lowering drugs that have anti-inflammatory activities and have been shown to reduce glial activation and increase blood-brain-barrier integrity (98). In a phase II clinical trial with 52 participants with mild TBI, atorvastatin administration for 7 days post-injury was safe but had no impact on neurological recovery (99) (NCT01013870). This phase II trial was not extended due to the inability to enroll sufficient numbers of patients. Another potential drawback of the trial was the timing of drug administration, which was allowed to be administered up to 24 h after injury, even though preclinical studies indicated treatment within the first few hours of injury is crucial for efficacy.

### Omega-3 Fatty Acid Treatment—Docosahexaenoic Acid

Docosahexaenoic acid (DHA) is an omega-3 fatty acid mainly found in fish oil which possesses anti-oxidative (100), anti-inflammatory (101, 102), and neuroprotective effects (103). DHA has been shown to reduce the level of endoplasmic reticulum stress and to inhibit abnormal protein accumulation [such as amyloid precursor protein (APP) and phospho-Tau proteins] within the brain following TBI (104). DHA treated rats did not show TBI induced autophagy biogenesis and had reduced TBI-induced hippocampal and cortical damage (105). In a rat pup brain injury model, DHA decreased oxidative stress and microglial-proinflammatory activation and improved short term cognitive function (106). In a recent clinical trial, DHA supplementation for 12 weeks after a mild TBI (the mean GCS for both DHA and placebo was >14) did not significantly affect the quality of life as assessed by changes in physical and psychosocial functioning (107) (NCT00671099). This study did not investigate injury markers or pathological changes. Another study assessed the effect of DHA on biomarkers of trauma in American Football players. DHA administration decreased serum NFL levels compared to placebo-treated athletes, but although the authors suggested that DHA supplementation is therefore neuroprotective, NFL was the only marker measured (108). A major limitation of this study is the inability to measure or control for factors such as hit severity and number of hits during the study period. This would increase the variability in severity between the different athletes and potentially impact their response to treatment.

### Gonadal Hormone Treatment

Estrogen and Progesterone are gonadal steroid hormones that have functions beyond roles in reproduction. Treatment with both hormones has shown neuroprotective effects in several diseases and injuries of the CNS, such as multiples sclerosis (109), stroke (110), and spinal cord injury (111). In rat models of TBI, estrogen promoted neuronal survival in the hippocampus and decreased neuronal degeneration in the hippocampus and cortex (112), and decreased brain edema (113, 114). And in a mouse and rat model of TBI, progesterone treatment reduced cerebral edema, apoptosis, and inflammation (115, 116). In a phase II clinical trial, progesterone treatment was given to 77 patients (vs. 23 placeboes), which included both severe and moderate injury classification. Overall, there was a strong trend toward fewer deaths when compared to placebo control. Progesterone treatment did not show promising improvement for severe patients (GCS 3–8) with regard to their Glasgow Outcome Scale-Extended and Disability Rating Scale scores. Moderate TBI (GCS 9–12) survivors treated with Progesterone were more likely to have a moderate to good outcome compared to the placebo-treated group (117) (NCT00048646). A phase III clinical trial extending this study, ProTECT III, was terminated due to a lack of demonstrable benefit (118) (NCT00822900). In another placebo-controlled clinical trial with 159 enrolled patients with a GCS ≤ 8 upon admission, progesterone treatment improved neurological outcome measurements at 3- and 6-months post-injury. The Progesterone treated group also had higher survival rates compared to the placebo group at 6 months (119). Only one clinical trial for Estrogen is listed in clinicaltrials.gov. The trial enrolled 48 TBI participants for Premarin IV treatment and was concluded in 2019, with no data currently available.

## Neuromyelitis Optica (NMO)

**Figure 3:**
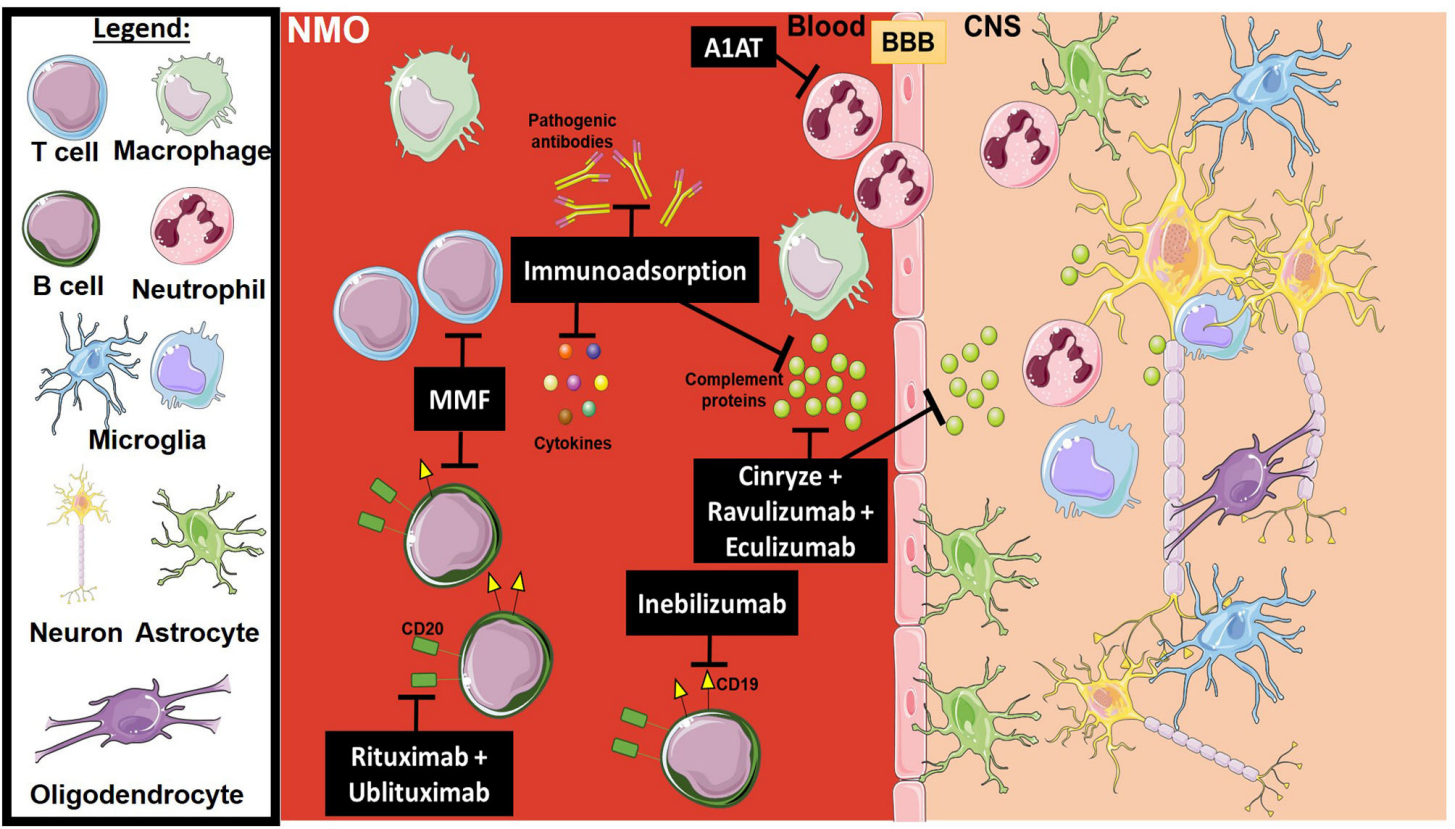
Mechanism of action for the therapeutics discussed in NMO section. Lines ending with a flat dash indicate an inhibitory effect, and lines ending with an arrow indicate a positive/stimulating effect. Cinryze (a C1-esterase inhibitor), Eculizumab (monoclonal antibody inhibitor against C5), and Ravulizumab (a second-generation monoclonal antibody inhibitor against C5) are all complement inhibitors. Anti-apoptotic (A1AT) inhibits plasminogen activators, as well as elastases which are important for neutrophil activation. Rituximab and Ublituximab are anti-CD20 monoclonal antibodies that target and eliminate B cells. Inebilizumab is an anti-CD19 monoclonal antibody that also targets B cells. MMF is an immunosuppressant that blocks the proliferation of many cell types, mainly B and T lymphocytes. Immunoadsorption and plasma exchange has multiple effects, including the elimination of activated complement proteins, pathogenic antibodies and pro-inflammatory cytokines. All drawings of cells/molecules used in this figure were obtained and modified from Servier Medical Art by Servier, licensed under a Creative Commons Attribution 3.0 Unported License (https://smart.servier.com/).

Neuromyelitis optica (NMO) spectrum disorders are a family of disorders characterized by autoimmune inflammation commonly affecting the spinal cord and optic nerve. NMO has considerable clinical and pathological overlap with MS, both of which are demyelinating autoimmune disorders, but is a distinct entity. NMO has a worse prognosis than MS, as attacks are more severe, and recovery from attacks is often incomplete (120). NMO is commonly associated with an antibody against aquaporin-4, which is expressed by astrocytes near the ventricles in the brain (121). This antibody activates complement and causes antibody-dependent cellular toxicity, contributing to paraventricular lesions in the brain (122). Current therapeutic options include steroids for acute exacerbations and chronic immunosuppression. Plasma exchange for removing pathologic antibodies is another approved therapeutic option. While these therapies are helpful in reducing inflammation or reducing the frequency of relapses, they have negative systemic side effects and carry a risk of infection. Treating NMO with a more targeted neuroprotective therapy may reduce some of these side effects and help prevent neuronal damage that occurs in this disease. Figure 3 provides a representative scheme of the mechanism of action for the discussed therapeutics in NMO.

### Complement Inhibition

The complement system plays a clear role in mediating damage in NMO via pathogenic autoantibodies, and there has therefore been interest in therapeutically inhibiting the complement system. A phase Ib clinical trial of the C1-esterase inhibitor Cinryze, which inhibits the C1 complex of the antibody-initiated classical complement pathway, was safe in humans, with no adverse events (123) (NCT01759602). However, the drug has not been studied further in NMO clinical trials, and a follow-up study in a rat model of NMO concluded that complement inhibition with this drug was too low to reduce pathology and to be of clinical benefit (124).

Eculizumab is an anti-C5 monoclonal antibody, and in a phase I and II clinical trials with 14 patients, the drug was shown to be well-tolerated, reduce attack frequency, and improved disability measures. However, one patient suffered meningococcal sepsis as a severe side effect (125) (NCT00904826). Following a phase III trial, eculizumab was approved by the FDA in 2019 to treat neuromyelitis optica in patients who test positive for anti-aquaporin-4 antibodies. In the phase III study involving 143 patients, the drug was shown to reduce the risk of relapse in combination with standard immunosuppression compared to placebo and immunosuppression. In the study, upper respiratory tract infections were more common in the eculizumab group, highlighting the risks associated with systemic complement inhibition and the need for monitoring patients receiving therapy (126) (NCT01892345). Eculizumab is currently undergoing a phase III clinical trial with pediatric NMO patients (NCT04155424), and Ravulizumab, a second-generation anti-C5 monoclonal antibody, is undergoing a phase III clinical trial with adult patients (NCT04201262).

The mechanism of classical complement pathway-mediated damage to autoantibody-targeted tissue in NMO bears some resemblance to myasthenia gravis (MG). While not specifically a CNS disease, MG is associated with complement-activating antibodies against components of the neuromuscular junction, most commonly the acetylcholine receptor. Eculizumab was approved for the treatment of MG in 2017 (127), and other inhibitors of C5 including Ravulizumab (NCT03920293) and Zilucoplan (NCT04225871) are undergoing clinical trials for MG.

### Anti-apoptotic

Alpha-1-Antitrypsin (A1AT) is a serine protease inhibitor (128) and is an FDA approved medication for several diseases, including A1AT Deficiency (129). It can inhibit plasminogen activators, chymotrypsin, as well as elastases, and can function as an anti-inflammatory and tissue repair molecule (130–132). In 2014, a non-randomized phase I clinical trial was initiated to investigate A1AT in NMO, using a single weekly dose of 120 mg/kg until a 4-dose regimen was completed, but the trial was withdrawn, and there have been no further updates (NCT02087813).

### Rituximab, Ublituximab, and Inebilizumab

Rituximab is a humanized glycosylated IgG antibody specific for CD20, a transmembrane protein expressed on both healthy and malignant B cells (133). Rituximab was approved by the FDA in 1997 and has been widely used in several autoimmune disorders, including: lupus, rheumatoid arthritis, and autoimmune anemia. A phase I clinical trial completed in 2010 analyzed the safety and tolerability of Rituximab in NMO patients (NCT00501748). Although enrollment was only 8 patients, 7 of the 8 had a decrease in neurological disability and remained symptom-free for up to 18 months. More recently, a multicenter, randomized, double-blinded, placebo-controlled clinical trial in Japan was conducted using Rituximab for patients suffering from NMO and were seropositive for aquaporin 4 (134) (UMIN000013453). Although the small sample size, treatment prevented relapses from occurring for up to 72 weeks in NMO patients. While these results are promising, additional clinical trials are needed to assess the safety and efficacy of this therapeutic agent in NMO patients.

Ublituximab is another monoclonal antibody that targets CD20, but with increased toxicity against malignant B-cells compared to Rituximab (135). A 2019 interventional clinical trial assessed the safety profile of Ublituximab as an adjunct therapy to steroids for the treatment of 5 NMO patients (136) (NCT02276963). A single intravenous dose of 450 mg resulted in successful B cell depletion within 8 weeks. Although there are numerous limitations to this clinical trial, there need to be more clinical trials that test Ublituximab's efficacy, as well as testing efficacy against a proper placebo group to determine this agent's impact in NMO patients. Ublituximab was safe in all 5 patients, with no serious adverse events reported and no opportunistic infections. Median Expanded Disability Status Scale (EDSS) scores dropped from 6.5 at admission to 4.0 at the 90-day follow-up. Two patients relapsed after not achieving complete B cell depletion. However, given that this study was only designed to assess safety, a full determination of efficacy will have to wait for a phase II study.

Inebilizumab is a monoclonal antibody that targets CD19, another protein expressed on B cells that is a viable therapeutic target independently of or in addition to CD20-targeting antibodies (137). Inebilizumab can also deplete plasma cells, while often express CD19 but not CD20. A 2014 phase 2/3 clinical trial comparing iv inebilizumab to placebo in 230 patients was ended early due to demonstration of efficacy. The inebilizumab-treated group showed a significant reduction in the risk of experiencing a relapse compared to placebo, and a similar adverse event profile (138) (NCT02200770). At the time of writing, this drug is still being reviewed for approval by the FDA.

### Immunosuppressants

Mycophenolate Mofetil (MMF) is an immunosuppressant that acts as a reversible inhibitor of inosine monophosphate dehydrogenase, an enzyme essential for synthesis of DNA nucleotides, which are important for cell proliferation (139, 140). In a retrospective case series study, patients treated with mycophenolate mofetil had a reduction in relapse frequency, coupled with stabilized or reduced disability (141). Similar results were detected in a clinical trial conducted in Korea (142). A phase IV trial of Mycophenolate Mofetil treatment in Southern China patients revealed that low dose of MMF resulted in a reduction in clinical relapse and disability (143) (NCT02809079). Although it is currently not FDA approved for NMO, it is commonly used off-label for it, to the extent that it is considered as a first-line of immunotherapies (144).

### Immunoadsorption/Plasma Exchange

Plasma exchange is a suggested therapeutic option used in NMO to prevent relapses (145). This treatment results in the elimination of pathogenic antibodies, complement components, and cytokines from the blood of patients suffering from NMO (146). A clinical trial carried out in India aimed at evaluating the efficacy of plasma exchange as a first line of treatment for acute attacks in NMO patients (147). Plasma exchange, especially if performed early after the first attack, resulted in a better outcome as assessed by percentage improvement in EDSS score. In the USA, a clinical trial is currently investigating plasma exchange in NMO patients, but no results have been reported (NCT01500681).

## Amyotrophic Lateral Sclerosis

Amyotrophic lateral sclerosis (ALS) is a fatal neurodegenerative disease characterized by motor neuron death. Both upper and lower motor neurons are affected. Progressive muscle atrophy and weakness eventually lead to death from the weakness of the respiratory muscles, with half of patients dying within 2.5 years (148). There is no cure for this disease, and the two approved medications only modestly improve survival. Riluzole was the first drug approved by the FDA for the treatment of ALS. It is a glutamate antagonist that reduces glutamate-associated toxicity to motor neurons, slowing the course of the disease (149, 150). Edaravone (Radicava), an antioxidant that protects motor neurons from oxidative stress, was approved by the FDA in 2017 (151). Both drugs help to slow the rate of physical decline in ALS patients through their neuroprotective function. Although these are the only other drugs currently approved for treating ALS, there are numerous other neuroprotective agents in clinical trials which are discussed below.

### NMDA Receptor Antagonist

Glutamate excitotoxicity is a mechanism by which damage occurs in motor neurons. Glutamate is the major excitatory neurotransmitter in the brain and spinal cord. Excessive concentrations of glutamate in the synaptic clefts, which can occur due to dysregulation of glutamate reuptake or excessive depolarization of the presynaptic cell, causes excessive depolarization of the post-synaptic cell. This depolarization causes an influx of calcium and leads to cellular swelling, activation of lytic enzymes, and eventually lysis, releasing cellular contents including glutamate and affecting nearby cells in a positive feedback loop. Riluzole acts through several mechanisms and inhibits glutamate release presynaptically, and activates receptors and channels post-synaptically (149, 152). In ALS, Riluzole exerts a neuroprotective effect in the spinal cord, where glutamate is used as a transmitter. The long history of the use of Riluzole to slow motor decline in ALS is an example of effective, albeit modest, neuroprotection. An early trial set out to determine the optimal dose of Riluzole to be administered in patients that were diagnosed with ALS in <5 years since onset (153). A 100 mg dose of Riluzole was determined as the optimal dose for the best benefit-to-risk ratio and improves survival rate.

### Antioxidants

Oxidative stress is a major contributor to the decline in synaptic function at the neuromuscular junction. Edaravone is a free radical scavenger and antioxidant initially developed by Mitsubishi Yuka Pharmaceutical Corporation for the treatment of ischemic stroke (154, 155). Its potential use in ALS was recognized, and three clinical trials were completed leading to its approval as a treatment for ALS (151, 156) (NCT00330681, NCT00415519, and NCT01492686).

### Iron Chelators

Oxidative stress can also be caused by free radicals generated by iron accumulation. Iron accumulation has been observed in several mouse models of ALS (157), as well as in post-mortem sections of motor tracts in ALS patients (158, 159). There is an ongoing phase III clinical trial with the iron chelator deferiprone in ALS with a projected end date of 2022 (NCT03293069). Deferiprone has been used for decades to treat iron overload with manageable side effects (160) and is also in other ongoing clinical trials for neurodegenerative diseases, including Alzheimer's disease (NCT03234686) and Parkinson's disease (NCT02728843).

### Mitochondrial Protection

Another therapeutically targetable mechanism of motor neuron distress is mitochondrial dysfunction. Oxidative stress, increased calcium, and cellular damage can induce pore formation in the mitochondrial membrane, leading to damage to and apoptosis of motor neurons. Olesoxime is a compound that binds to two outer mitochondrial membrane proteins associated with mitochondrial response to oxidative stress (161). Olesoxime is neuroprotective for rat neurons deprived of trophic factors *in vitro* (162) and was shown to improve motor performance and survival in an ALS mouse model. However, it failed two clinical trials as an add-on therapy for Riluzole for ALS (did not show a survival benefit) (163) (NCT00868166 and NCT01285583). It also failed to prevent a decline in motor function in clinical trials for spinal muscular atrophy (164) (NCT02628743 and NCT01302600). Preclinical studies with olesoxime showed it exerts its greatest protective effects on neuromuscular junctions and glial activation when administered before symptom onset (165), which may explain why a beneficial effect was not observed in ALS patients. Olesoxime is metabolized in a similar manner to cholesterol, so variability in cholesterol metabolism in patients may explain the high variation in bioavailability of olesoxime (163). Tauroursodeoxycholic acid (TUDCA) is another mitoprotective agent in clinical trials in ALS. TUDCA was originally developed to treat cholestatic liver disease due to its structural similarities to bile acid. However, it has also been shown to be anti-apoptotic via its interaction with mitochondria. It inhibits apoptosis by stabilizing the mitochondrial membrane and inhibiting the translocation of the pro-apoptotic protein, Bax, from the cell to the mitochondria (166). This finding has led to an interest in the compound as a treatment for various other neurodegenerative diseases in addition to ALS. TUDCA was shown to be safe for ALS (167) (NCT00877604) and is currently in a phase III clinical trial for ALS (NCT03800524).

### Clearance of Protein Aggregates

The accumulation of toxic levels of protein aggregates is a common feature of neurodegenerative disorders and is seen in other disorders such as Alzheimer's disease, Parkinson's disease, and Huntington disease. In ALS, misfolded aggregates of the proteins TDP-43 (168) or SOD1 (169) in neurons contributes to neuronal death. Ibudilast is a phosphodiesterase 4 inhibitor that, among other things, enhances autophagy of protein aggregates through inhibiting mTORC1 activity, and protects motor neuron-like cells from TDP-43 induced cytotoxicity (170). Ibudilast is currently undergoing a phase IIb/3 clinical trial as an add-on for Riluzole for ALS (NCT04057898) and a phase I/II clinical trial as a stand-alone agent (NCT02714036). Results from a smaller phase II clinical trial for Ibudilast (NCT02238626) show that Ibudilast together with Riluzole reduces ALS disease progression relative to Riluzole alone; however, this effect was noted only in patients with a short (<600 day) history of ALS, and differences in baseline duration of ALS between treatment and placebo groups confound the results. The results of the phase IIb/III clinical trial will help clarify this result.

### Complement Inhibition

Activation of the complement system is associated with neuronal damage and inflammation in ALS. Complement deposition has been observed at the neuromuscular junction in ALS patients (171), and C5a and the MAC are elevated in ALS patient blood (172). Preclinical murine studies have shown benefit when inhibiting C5a receptor 1 (C5aR1) with the experimental drug PMX205 (173, 174), or inhibiting the MAC with a C6 RNA antagonist (175). Several recent reviews have described the role of complement in the pathology of ALS and the possible therapeutic benefit of targeting complement (176–178). Two clinical trials have been announced recently that investigate complement inhibition in ALS, both at the level of C5. Alexion Pharmaceuticals has announced a phase III clinical trial of Ravulizumab that plans to enroll 354 participants (NCT04248465). Ra Pharmaceuticals has announced a phase II/III clinical trial of Zilucoplan, a synthetic peptide inhibitor of C5, with a planned enrollment of 480 participants (NCT04297683).

## Multiple Sclerosis

Multiple sclerosis (MS) is a common autoimmune inflammatory disorder that can affect multiple parts of the central nervous system. The exact cause of MS is unknown, but the disease is the complex interplay of genetic factors, such as specific human leukocyte antigen (HLA) alleles or other polymorphisms and environmental factors, such as vitamin D levels, smoking, or certain viral infections (179). The disease is characterized by acute neurological episodes in which blood-brain barrier integrity is compromised, with cellular and molecular components of the immune system infiltrating focal areas of the CNS and contributing to demyelination (180). Common symptoms of an MS episode are visual deficits or eye pain due to optic neuritis, neuropathies or myelopathies due to spinal cord involvement, and ophthalmoplegia or nystagmus due to the involvement of different myelinated tracts (181). Patients can experience complete or partial recovery between episodes, with recovery attributed to a decrease in acute inflammation and ongoing remyelination. The goal of many therapies of MS is to reduce the incidence or severity of relapses. MS can be roughly divided into four subtypes, each with different etiologies and disease courses, but with similar symptoms. The most common subtype of MS, and for which the majority of approved therapies are for, is relapsing-remitting MS (RRMS) (182). There are several therapies for MS currently in clinical trials.

### Antioxidant Therapeutics

Precise details of inflammatory cascades involved in MS pathogenesis remain unclear, but reactive oxygen and nitrogen species, as well as pro-inflammatory cytokines produced by infiltrating macrophages, have been strongly implicated in demyelination and axonal damage (183). Experimental models of MS show a protective effect of antioxidants (184), and several antioxidants have been tested in experimental models and in clinical trials.

Polyphenon E is a green tea extract (Camellia sinensis) with the active ingredient Epigallocatechin gallate (EGCG), a mitochondrial antioxidant that helps reduce oxidative stress and cell death (185). In experimental autoimmune encephalomyelitis (EAE) models of MS, EGCG was shown to have a neuroprotective effect (186, 187), which led to clinical trial trials (NCT00836719 and NCT01451723). However, the phase II trial was terminated early due to hepatotoxicity within a large number of participants (188).

Glutathione (GSH) is an endogenous antioxidant that protects cells from oxidative stress (189, 190), and reduced glutathione concentration and a simultaneous decrease in alpha-tocopherol levels in MS patients provided early evidence of elevated ROS during the active disease state (191). Challenges to GSH administration included solubility, absorption, and stability, thus limiting its practical use (192). Delivery of the precursor cysteine resulted in significant side effects (193). An alternative approach is to target the pathway of GSH synthesis. Activation of the nuclear factor (erythroid-derived 2)-2 (Nrf2) pathway is involved in the regulation of most enzymes necessary for GSH synthesis (194), and Dimethyl fumarate (DMF), an indirect activator of Nrf2, has shown promising results. Multiple phase II and III studies have reported a reduction in relapse episodes, as well as lesion number and size in MS patients (195) (NCT00835770, NCT00420212, NCT00451451, and NCT02047097).

Epidemiologic evidence suggests vitamin D involvement in MS progression. High levels of 25-hydroxyvitamin D [25(OH)D] have been associated with lower MS risk, and decreased risk of MS has been studied among offspring of mothers who had high 25(OH)D levels (196). Experimentally, vitamin D administration in an EAE model resulted in slowed disease progression with modulation of T-helper 17 (Th17) cell differentiation and interleukin-17a (IL-17a) expression (197). However, clinical trials failed to provide conclusive evidence that vitamin D slowed disease progression (198) (NCT00785473, NCT01339676). A possible reason for this may be related to causation vs. correlation. 25(OH)D is converted to calcitriol by 1-alpha-hydroxylase, an enzyme encoded by the CYP27B1 gene. Mutations in this gene have been found to be transmitted from heterozygous parents to MS offspring (199), and the relationship between 25(OH)D levels and MS disease progression may simply be due to multi-functionality of the same gene encoding the MS trait as well as vitamin D processing.

### Neurotransmitter Modifiers

MS has long been known to be associated with neurochemical alterations in and outside of the CNS. Excessive extracellular accumulation of excitatory neurotransmitters, namely glutamate and aspartate, have been directly correlated with disease severity and neurologic deficits (200, 201). Multiple neurotransmitter-altering agents have been investigated in clinical trials for MS, including Riluzole, Fluoxetine, Memantine, Rivastigmine, and D-aspartate.

In a large clinical trial (MS-SMART) evaluating multiple agents in secondary progressive MS that included Amiloride (sodium channel blocker), Fluoxetine (a selective serotonin reuptake inhibitor), and Riluzole (glutamate receptor antagonist), there was no evidence of neuroprotection in any patient group compared to placebo, possibly due to low relevance of the pathways targeted (202) (NCT01910259). Memantine, an uncompetitive antagonist of NMDA-type glutamate receptor (see above), was tested in a randomized controlled trial of RRMS patients with cognitive impairment and found to have no effect on memory or cognition in patients with MS-related dementia; there were also significant neurological side-effects (203) (NCT01074619). Another agent tested for cognitive impairment in MS is Rivastigmine, an acetylcholinesterase inhibitor that has previously shown positive results in cognitive function in patients with Alzheimer's disease. Multiple clinical trials were conducted, with several showing low tolerance for side effects, while others showed no clinical efficacy in preventing disease progression of MS-related dementia. A tendency toward improvement in total recall was recorded, but low enrollment led to insufficient statistical power to demonstrate a significant effect, and other tests of cognition suggested no effect of rivastigmine relative to placebo (204, 205) (NCT00881205).

D-aspartate is an amino acid that can be localized to multiple brain regions and is used for the production of various hormones like gonadotropin-releasing hormones, luteinizing hormone, testosterone, and melatonin (206–208). A recently completed clinical study in Italy demonstrated positive effects of D-aspartate on neuroplasticity measured by trans-magnetic stimulation (TMS), suggesting further clinical studies are warranted (209).

### Sodium Channel Blockers

Axonal sodium overload due to conduction block from nitric oxide is also implicated in neuronal injury during the degenerative process in MS (210–212), and an *in vitro* study demonstrated that partial blockade of sodium channels reduced axonal damage induced by persistent activation of sodium channels that led to an accumulation of extracellular ions (213). Sodium channel blockers have been used clinically for many years for seizure control and spasticity. Here we discuss sodium channel blockers that have been examined in the setting of MS.

A clinical trial for the sodium channel blocker, Lamotrigine, with an intention-to-treat design that ultimately enrolled 108 patients with placebo and treatment did not find evidence of neuroprotection or clinical improvement (214) (NCT00257855). Of note, the dose in an EAE model was 30 mg/kg, whereas dose in this clinical trial was 78 mg or 1.1 mg/kg for an average 70 kg human. However, elevated doses of this class of drugs can have severe side effects and neuronal disturbance and can be fatal.

Other agents such as carbamazepine and phenytoin are not as well-studied in MS. However, small scale clinical studies with carbamazepine were conducted to evaluate efficacy for paroxysmal disorders within MS, such as trigeminal neuralgia (215, 216). Higher doses were shown to have low efficacy, but with significant side effects. Phenytoin was studied in acute demyelinating optic neuritis, a feature of MS in which damage to vision occurs through optic nerve degeneration. In a small clinical study, phenytoin reduced the rate of loss of retinal nerve fiber layer thickness in affected eyes, suggesting a neuroprotective effect. However, no clinical benefit was found (217) (NCT01451593).

A common challenge with sodium channel blockers is the relatively high doses necessary for efficacy, which is accompanied by significant side effects and which in general precludes their use for promoting neuroprotection. They nevertheless remain common drugs for the treatment of spasticity and seizures.

### Immunomodulation

Multiple sclerosis is an autoimmune disease, and many approved therapies and several current clinical trials focus on suppressing the immune response to reduce the severity of neurological episodes. Interferon beta was approved by the FDA in 1993 for MS (218). While its exact mechanism of action is unknown, it skews both T cell activity and cytokine profiles toward an anti-inflammatory phenotype (219). Corticosteroids, such as methylprednisolone, are used during acute exacerbation of MS to reduce the severity of episodes; they exert multiple anti-inflammatory effects, including reducing infiltration of inflammatory cells into the CNS and possibly inducing apoptosis (220). Adrenocorticotrophic hormone, which stimulates the endogenous production of corticosteroids, was approved by the FDA in 1978 (221). However, oral or intravenous steroids have superior efficacy in treating relapses (222) and are the standard of care for acute relapses.

Glatiramer acetate is an amino acid polymer with a similar structure to myelin basic protein, which is a known autoimmune target in MS. Due to this structural similarity, it may compete with myelin binding to antigen-presenting cells and suppress autoimmunity and induce an anti-inflammatory response. It also contributes to remyelination by inducing the secretion of neurotrophic factors from myelin-reactive T cells. An early phase III clinical trial using the glatiramer acetate, Copolymer1, resulted in a decreased relapse rate over a 2-year period, which was coupled with improved disability (223). Glatiramer acetate was approved by the FDA in 2018 for the treatment of RRMS (224).

Other immunomodulatory agents have been under investigation for MS. Methotrexate is an antimetabolite commonly used for rheumatoid arthritis and certain cancers. It inhibits nucleic acid synthesis, causing preferential toxicity in rapidly dividing cells such as lymphocytes. Methotrexate has been investigated in RRMS in several clinical trials (225) (NCT00037102, NCT00112034, and NCT00037115) and has efficacy similar to that of interferon beta-1a (226). Intrathecal methotrexate has also been shown to be safe in treating progressive forms of MS (227) (NCT02644044). Methotrexate is not currently FDA approved for treating either form of MS but is sometimes used off label as a second-line agent.

Minocycline is an antibiotic that exerts anti-inflammatory and neuroprotective effects (see above). Minocycline crosses the blood-brain barrier and modulates T cell behavior, reduces microglial activation, and prevents neuronal apoptosis (228). It has been the subject of several clinical trials for RRMS (NCT01134627, NCT04291456, NCT00203112, and NCT00666887), although with unclear results. Completed trials suggested a trend toward lower relapse rates (229) or lower risk of conversion of clinically isolated syndrome (CIS) to MS in the short term (230), but more studies are needed. In addition, Minocycline combined with administration of subcutaneous interferon-beta 1a showed no statistically significant benefit for MS patients (231).

### Targeted Immunotherapy

There are several approved immunotherapy options for MS which differ from immunomodulation in that they are antibodies that have direct molecular targets, rather than compounds that exert multiple effects on the immune system.

One modality is anti-B cell or anti-lymphocyte antibodies, which are commonly used in treating RRMS and primary-progressive MS PPMS (232). These antibodies target mature B cells (CD20) or B and T cells (CD52). Ocrelizumab and Rituximab are two anti-CD20 antibodies used for the treatment of MS. Anti-B cell reagents such as these have been shown to reduce the relapse rate and presence of new lesions in the CNS (233). In several identically designed phase III studies, Ocrelizumab showed efficacy in treatment when compared to interferon-beta 1a with regard to improved outcomes in areas such as disability progression and suppression of new inflammatory lesions in the brain detected by magnetic resonance imaging (MRI) (234) (NCT01247324, NCT01194570, and NCT01412333). Similarly, Rituximab administration reduced inflammatory brain lesions along with a decrease in clinical relapses for 48 weeks (235) (NCT00097188). Alemtuzumab is an anti-CD52 antibody approved for RRMS that depletes B and T cells, with significant clinical benefit when compared to interferon beta-1a (236) (NCT00050778, NCT00530348, and NCT00548405). Natalizumab is another approved monoclonal antibody for MS that targets alpha4 integrin expressed on the surface of activated lymphocytes. This molecule is part of a receptor that interacts with vascular endothelium in the brain, tethering lymphocytes to the endothelium and allowing them to migrate through the blood-brain barrier. Natalizumab blocks this interaction, reducing the severity of relapses (237). In a phase III trial, Natalizumab resulted in visual improvement in relapsing MS patients (238) (NCT00027300). Natalizumab was approved by the FDA in 2007 after showing a reduction in relapse rate and severity (239). Of note, a particularly severe side effect of natalizumab therapy is progressive multifocal leukoencephalopathy, a fatal disorder caused by reactivation of the John Cunningham (JC) polyomavirus. Therefore, patients are screened for antibodies against JC virus before initiating therapy (237).

## Parkinson's Disease

**Figure 4:**
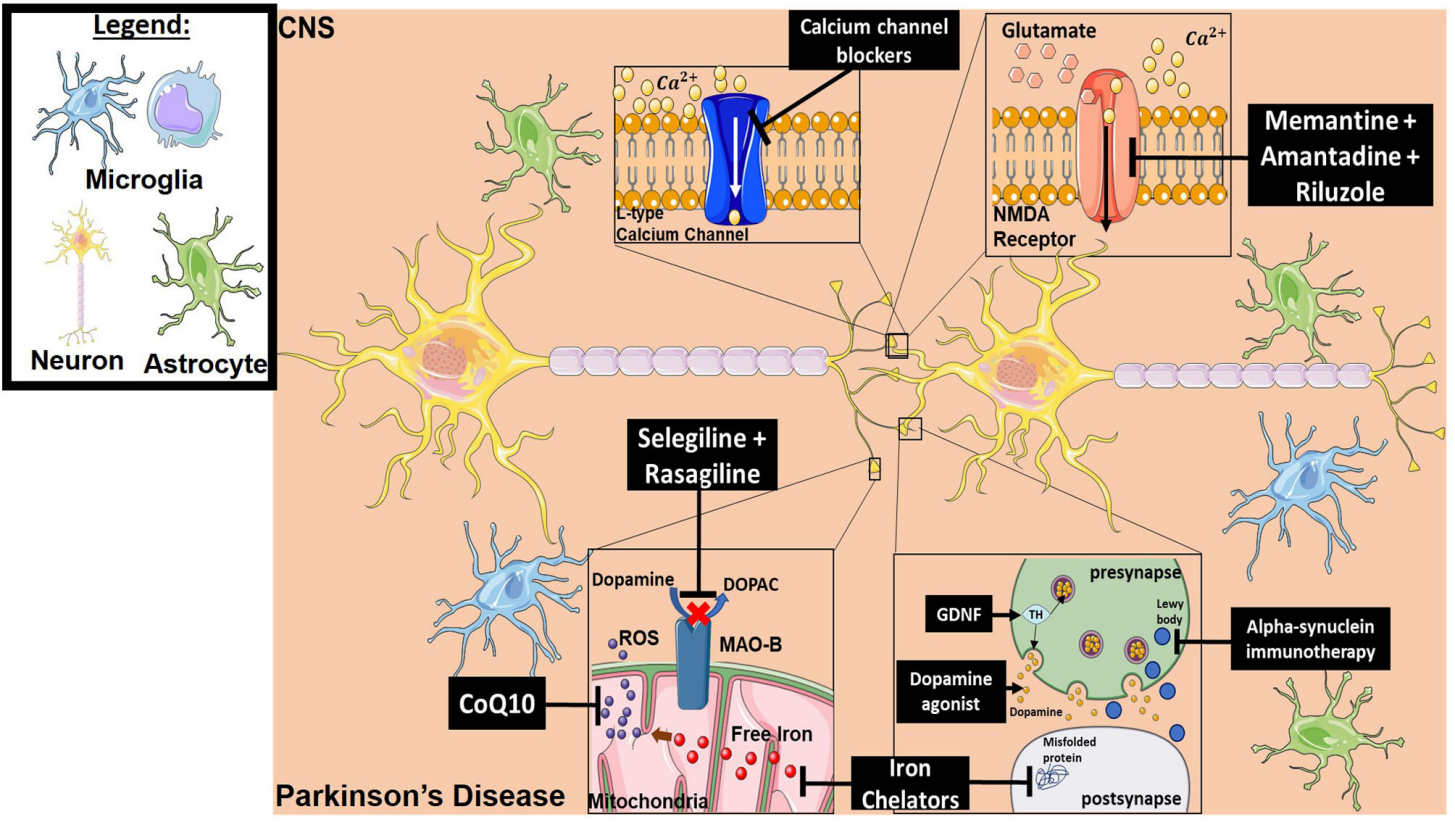
Mechanism of action for the therapeutics discussed in PD section. Lines ending with a flat dash indicate an inhibitory effect, and lines ending with an arrow indicate a positive/stimulating effect. Dopamine agonists function to provide dopamine-mediating synaptic function which is lost in PD. They are either precursors of dopamine to ensure production, or dopamine receptor agonists to ensure signaling. Iron chelators inhibit both free iron production (which will result in ROS generation) and misfolded protein formation. Alpha-synuclein immunotherapy decreases the formation of Lewy bodies resulting from aggregation of alpha-synuclein. Neurotrophic factor treatment (such as GDNF) provides tyrosine hydroxylase which in turn results in dopamine production. CoQ10 is an antioxidant and ROS scavenger. Selegiline and Rasagiline are monamine oxidase B inhibitors that prevent dopamine from converting into DOPAC, which can then be oxidized and forms toxic metabolites. Calcium channel blockers prevent intracellular calcium dysregulation. Memantine, Amantadine and Riluzole have an inhibitory effect on NMDA receptors, which reduces excitotoxicity. All drawings of cells/molecules used in this figure were obtained and modified from Servier Medical Art by Servier, licensed under a Creative Commons Attribution 3.0 Unported License (https://smart.servier.com/).

Parkinson's disease (PD) is one of the most common neurodegenerative disorders globally (240). It is a complex pathology that is primarily driven by progressive neuronal cell loss within the substantia nigra (241). At diagnosis, it is estimated that almost a 3rd of dopaminergic neurons are already lost (242). A common theme underlying PD is a generalized inflammatory response that leads to the accumulation of alpha-synuclein aggregates and reactive oxygen intermediates, ultimately resulting in dopaminergic cell loss (243, 244). Factors thought to be involved in PD progression include Monoamine oxidase B (MAO-B) that degrades dopamine, iron accumulation, protein aggregation, glutamatergic excitotoxicity, altered calcium gradients, and perpetual neuroinflammation. There are currently no curative treatments for PD, but the most effective drugs for symptom control are dopaminergic agonists such as Levodopa (242). In the context of neuroinflammation, multiple classes of drugs have been developed that interfere with inflammatory processes known to be involved in disease pathogenesis. Below we discuss several classes of neuroprotectants that have been investigated for the treatment of PD. Of note, however, no neuroprotectant has yet shown convincing efficacy in the clinic, although this is an active area of current research in PD treatment (245). Figure 4 provides a representative scheme of the mechanism of action for the discussed therapeutics in PD.

### Dopamine Agonists

Dopaminergic neurons are the primary cells lost in the inflammatory cascade in PD. Dopaminergic agonists have been the gold standard for treatment and symptomatic delay of patients with PD. Primary drugs in this class used to treat PD include Levodopa (dopamine precursor), bromocriptine (D2 receptor agonist) ropinirole and pramipexole (D2 and D3 receptor agonist), and R-apomorphine (D1 and D2 receptor agonist). These agents are thought to decrease nigrostriatal cell loss following dopaminergic auto-receptor stimulation (246–249). Drugs of this class have been in multiple clinical trials, and many have shown some symptomatic improvement of patients (250–253) (NCT00804479 and NCT00004733). In a randomized delayed-start trial, neither early nor late administration of the dopamine agonist pramipexole had any disease-modifying effects or improvements in PD patients (254) (NCT00321854). A likely contributing factor to the failure if this trial is the high dose required for pramipexole to show clinical efficacy (noted at 1.5 mg per day). At this dose, the study showed ~10% severe adverse effects and over 80% general adverse effects. This is in contrast to pre-clinical studies that utilized doses of 1 mg/kg/day (255). Similar results were obtained in another clinical trial that administered Levodopa at early and late time points after PD diagnosis (256) (Trial Number: ISRCTN30518857). Taken altogether, it remains unclear whether this class of therapeutic provides definitive neuroprotection or simply a symptomatic delay effect without a clear disease-modifying effect (250, 256).

### Monoamine Oxidase B Inhibitors

Monoamine oxidase B inhibitors such as Selegiline and Rasagiline were developed to target the MPTP pathway (refer to TBI section above). These inhibitors have been used as an adjuvant that allows dosage reduction of dopamine agonists (levodopa), although recent data suggests that selegiline may have neuroprotective properties as a monotherapy (257). Large multi-center trials have shown a modest symptom delay when using selegiline compared to placebo, although it is unclear if this is due to a neuroprotective mechanism (258–264). On the other hand, Rasagiline was found to provide greater therapeutic benefit than Selegiline, with a higher antioxidant effect on peroxynitrite, MPTP, and 6-OHDA metabolism. One clinical trial using Rasagiline at 1 or 2 mg/day showed that the latter did not show any disease-modifying effect, but the 1 mg/day dose did provided benefits of a possible disease modifying effect using the Unified Parkinson's Disease Rating Scale (UPDRS) (265) (NCT00256204). However, a few years later, a large retrospective real-life study revealed no difference between Selegiline and Rasagiline in disease progression and time to levodopa (266). A potential pitfall for these trials is the assumption of a primary inflammatory pathway in PD, thus rationalizing monotherapy with Rasagiline as a neuroprotectant when a multi-therapy cocktail approach may be optimal.

### NMDA Receptor Antagonists

Although glutamate is not the primary neurotransmitter implicated in PD, alterations of glutamatergic transmission contribute to the disease process through excitotoxicity (267, 268). NMDA glutamate receptor is an excitatory, ligand-gated ion channel composed of multiple subunits. NMDA receptors have multiple regulatory properties, and their presence in the striatum is essential for dopamine-glutamate interaction. Their number and function are modified by dopamine depletion, as well as by therapeutics that are used to treat PD (269, 270). The primary goal of NMDA antagonism in PD is to ameliorate Parkinsonian symptoms such as dyskinesia, as well as to reduce the long-term effects of dopamine-based PD treatments (levodopa) (269). Several agents have been developed for non-targeted NMDA inhibition including Amantadine (a nicotine and NMDA antagonist originally used as an antiviral medication) (271), Memantine (a non-competitive NMDA receptor antagonist), and Riluzole (primarily a sodium channel blocker with an indirect blockade effect on NMDA receptors) (149). Pre-clinical studies with Riluzole showed a promising protective effect on dopaminergic cells in the MPTP toxin model, but in clinical trials, Riluzole failed as a treatment modality for PD (264, 272, 273). This may be due to the broad effect of Riluzole on multiple receptors. Amantadine also had a modest effect on slowing disease progression in the MPTP animal model, and clinical studies demonstrated a protective effect (274–276). One of the first clinical trials with Amantadine was published in 1970 and showed modest neuroprotection for patients with various stages of PD, with a well-tolerated side effect profile (277). More trials have since shown temporary improvement in motor complications and reduced dyskinesia (278–280) (UMIN Clinical Trial Registry UMIN000000780). Most recently, the EASE LID study (NCT02136914) evaluated extended-release amantadine capsules and found significant efficacy in the reduction of dyskinesia with a more consistent daily effect (281, 282). Although using a relatively small sample size, memantine has been shown to improve PD symptoms, specifically PD-associated dementia (283) (Trial registry number ISRCTN89624516). On the other hand, in contradiction to this report, Emre et al. demonstrated that memantine administration to patients with mild to moderate PD-dementia or dementia with Lewy bodies (DLB) showed no significant improvement in the PD-dementia group and a possible clinical benefit in the DLB group (284) (NCT00855686). Other studies demonstrated a significant deterioration following the discontinuation of the agent, indicating a symptom-control mechanism rather than a neuroprotective effect (285).

### Iron Chelators

Iron deposition in the substantia nigra pars compacta is implicated in PD progression via oxidative stress and protein misfolding that ultimately results in Lewy body deposition (286, 287). Iron chelators such as deferoxamine and phytic acid have had positive results in preclinical models, but clinical studies have not indicated any neuroprotective properties (288). Another iron chelator, deferiprone, was tested in a recent randomized controlled trial, and although it decreased iron content in the dentate and caudate nuclei, it did not provide significant clinical benefit (289) (NCT01539837). In another trial, investigators showed benefit of deferiprone treatment after 6 months of diagnosis translated by an improved UPDRS score when treatment was started early compared to a delayed treatment (290) (NCT00943748).

There are currently over 15 clinical trials investigating iron chelation for the treatment of neurodegenerative diseases, five of which are for PD (291). To date, clinical data remain inconclusive. A potential reason for the lack of success of iron chelation in the treatment of PD to date may be related to a dual role of iron in the basal ganglia and substantia nigra. Although its presence is associated with disease progression, iron is also necessary for energy production and dopamine synthesis, although the byproduct of this process is the redox-active form that ultimately results in the production of hydroxyl radicals (292). Another potential explanation, and which may be applied to several types of therapeutic under investigation, is that due to the multifactorial nature of PD, a single target is not adequate to induce significant neuroprotective effects.

### Calcium Channel Blockers

L-type calcium channel (LTCC) blockers showed efficacy in both MPTP and 6-OHDA animal models of PD (293, 294). The rationale for calcium-based treatment stems from large epidemiologic studies that showed a reduced risk of developing PD in patients taking blood-brain barrier-permeable calcium channel blockers for hypertension (295–299). *In vitro* and *in vivo* studies demonstrated that LTCC-mediated Ca^2+^ entry into substantia nigra dopaminergic neurons resulted in high metabolic stress levels and an increase in PD stressors and cell death (300, 301). Additional studies revealed a LTCC-mediated balance between protective and degenerative signaling in substantia nigra dopaminergic (DA) neurons, with a PD stressor increase tipping the balance toward degenerative signaling (302–305). The current theory is that LTCC blockers restore the balance and reduce degenerative signaling to maintain a protective microenvironment (306). A large clinical trial evaluated the LTCC blocker Israpidine in patients with early PD (NCT02168842). Initial findings from the trial revealed no benefit relative to placebo (307), and the authors speculated that the drug dose was insufficient to engage target calcium channels to exert a neuroprotective effect. An additional challenge in developing a LTCC blocker is the similarity amongst different calcium channel subtypes, including T and N-type, and the potential for creating an unwanted global effect (308).

### Neurotrophic Factors

Effects of growth factors on neurodegenerative disease modification have been extensively studied in preclinical models (250). In the MPTP animal model of PD, glial-derived neurotrophic factor (GDNF) increased the number and size of tyrosine hydroxylase (TH)-positive cells, indicating DA neuronal presence (309). An initial clinical study of intra-putaminal GDNF yielded a 39% improvement in the off-medication motor sub-score of the UPDRS, as well as a 64% reduction in dyskinesias (310). However, other studies investigating intraventricular or intra-putaminal administration did not result in significant clinical improvement (311–313). A likely cause of failure is the lack of blood brain barrier (BBB) penetrance for GDNF in the intraventricular groups. Furthermore, adequate putaminal administration would require significantly higher doses given the size of the target to confer clinical efficacy.

### Coenzyme Q10

Coenzyme Q10 (CoQ10) is an antioxidant present within the mitochondrial respiratory chain and functions as a ROS scavenger (314). As such, CoQ10 has been investigated as a treatment for PD, since disease progression is linked to mitochondrial defects and oxidative stress (315). An early study that randomized patients to multiple doses showed some short-term improvement and lower deterioration in clinical function at very high doses (1,200 mg/d) (316). However, subsequent and larger clinical studies failed to show any benefit in patients treated with Coenzyme Q (317–319) (NCT00180037 and NCT00740714), although some studies did suggest a mild symptomatic relief due to its alternative effect as an anti-depressive agent (316). Furthermore, a neuroprotective effect could not be determined since functional testing was performed while CoQ10 was present in patient serum, without confirmation of continued improvement following systemic decline of drug levels (318).

### Alpha-Synuclein Immunotherapy

Alpha-synuclein (alpha-syn) immunotherapy has emerged as a novel approach for the treatment of PD. Alpha-syn is a neuronal protein that is expressed in the presynaptic terminal and is involved in synaptic regulation (320). Alpha-syn protein accumulates extracellularly and forms Lewy bodies that are associated with PD dementia (320). There is an especially strong link between alpha-syn accumulation and PD (321). There are several known genetic mutations in the alpha-syn (SNCA) gene that are linked to both familial and sporadic forms of PD (322, 323). In a transgenic mouse model of PD, administration of recombinant alpha-syn protein generated antibodies that resulted in reduced behavioral deficits and reduced alpha-syn deposition (324). A monoclonal antibody has also been developed that recognizes an epitope for the C-terminal part of alpha-synuclein, causing decreased overall accumulation of alpha-syn aggregates and improving functional deficits in the mouse model, but no clinical studies have been conducted (325). A major challenge facing the successful translation of an anti-alpha-synuclein immunotherapy is the lack of specificity to pathologic alpha-syn (326). Also, the lack of BBB permeability would likely be an obstacle that could possibly be overcome by antibody engineering. Nevertheless, several clinical trials are underway to evaluate the efficacy of this therapy in PD patients. Active immunity drugs such as PD01A and PD03A, which are peptides mimicking c-terminus portions of the alpha-synuclein protein, have undergone phase I randomized controlled trials; they were tolerated at administered doses, but clinical data has not yet been published (327). Passive immunity drugs tested include PRX002, an anti-alpha-synuclein antibody, which was well-tolerated in humans and is currently undergoing a phase II clinical trial (328).

## Summary and Discussion

To date, neuroprotective therapeutics have, in general, provided disappointing results in clinical trials, despite the fact that most of the reagents investigated in the clinic showed promise in preclinical studies. There nevertheless continues to be optimism and a significant interest in the development of neuroprotectants for the treatment of CNS injury and disease. There are many potential explanations for the past failures of neuroprotectants in clinical trials. One such explanation is the selection of a drug candidate for clinical trials based on data from inappropriately designed preclinical studies, at least with regard to translation. Taking stroke as a case in point, potential reasons that preclinical success did not translate include: Treatments given too early in preclinical models, which does not translate; a focus on acute outcomes that do not account for chronic recovery; focus on gray matter injury without account for white matter injury; no comparison of rehabilitation-induced (standard of care) or spontaneous recovery; lack of account for gender and age-related effects. And of course, animal models are more homogenous in terms of reproducibility of injury mechanism and response to treatments compared to the heterogenicity of a patient population.

Other key considerations that can contribute to failure or difficulty in interpreting data include poor patient enrollment, especially when trying to match patients with comparable comorbidities, resulting in a vastly heterogeneous study population. In TBI trials for example, enrollment conditions for severity have, in general, not been optimal. Many studies enrolled individuals who had a GCS ≤ 8, but the nature of the injury is not uniform for all patients who receive a GCS ≤ 8. For example, a patient assigned a score of 4 with a pathologic reflex and no optical response is very different from a patient with a score of 7 and a purposeful reflex and optical response. Also, some clinical studies have used only one or two blood markers as a measure of treatment efficacy, and where possible, including more biomarkers would provide a broader and more conclusive outcome determination. Another consideration is that many of the therapeutics investigated, or that will be investigated, have systemic activity and may have dual function within the CNS vs. periphery, and which may have detrimental off-target effects which mask or render irrelevant on target benefits.

At the time of writing, complement inhibition has not been investigated in the clinic for MS, PD or TBI. We nevertheless included these diseases/injuries in this review because of the strong preclinical evidence indicating a role for complement in propagating pathology. For example, in the experimental autoimmune encephalomyelitis mouse model of MS, inhibition of C3 (329, 330), the alternative pathway (330, 331), or the terminal MAC provides protection against chronic disease and reduces neurological disability. Inhibition of C3 and specifically the alternative pathway protects against acute (332) and chronic (23) injury after TBI, and many other preclinical studies have demonstrated the effectiveness of complement inhibition in TBI [reviewed in (333–336)]. While no preclinical studies have investigated complement inhibition in PD models, complement components C1q (337) and C3 (338) deposit in the substantia nigra in PD. Also, the absence of complement receptor 3 (CR3) reduces loss of dopaminergic neurons in the paraquat and maneb-induced PD model (339), and the absence of C3 reduces loss of the dopaminergic neurons in the lipopolysaccharide challenge PD model (340). Thus, these preclinical studies suggest a role for complement in the pathogenesis of PD, although further study is needed.

Looking forward, an optimum candidate may be a multi-target, multi-pathway acting therapeutic that can selectively target injurious mechanisms while allowing for recovery. In this context, the complement system plays a central role in inflammation and modulates multiple downstream pathways once activated. Over the past several years, there has been explosive growth in both academic and commercial programs aimed at developing complement inhibitory drugs, with many anti-complement therapeutics now in various stages of clinical trials. Considering the evidence that complement plays key roles in driving multiple CNS pathologies [reviewed in (22, 177, 341–343)], diseases of the CNS would appear to be an attractive and viable target indication for the next generation of complement therapeutics.

## Author Contributions

KM and ST were responsible for conceptualization of the review topic. KM, CC, DB, MA, AT, and ST contributed to the writing of the manuscript. All authors contributed to reviewing the manuscript.

## Conflict of Interest

ST is cofounder and consultant for Q32Bio, a company developing complement inhibitors. The remaining authors declare that the research was conducted in the absence of any commercial or financial relationships that could be construed as a potential conflict of interest.

## Supplementary Material

Supplementary Table 1 This table summarizes the different clinical trials discussed in the manuscript. To date, according to clinicalTrials.gov, 458 of the 4,037 interventional clinical trials in stroke are/were in phase III (11.35%), 80 of the 875 interventional clinical trials in TBI are/were in phase III (9.14%), 75 of the 485 interventional clinical trials in ALS are/were in phase III (15.46%), 286 of the 1,596 interventional clinical trials in MS are/were in phase III (17.92%), 13 of the 44 interventional clinical trials in NMO are in phase III (29.54%), and 274 of the 2,031 interventional clinical trials in PD are in phase III (13.49%). Primary Outcome Met: Y, Yes; N, No; U, Unfound; W, Worse. All Outcomes: SB, Significantly Better outcomes with treatment; SW, Significantly Worse outcomes with treatment; NS, No Significant difference between treatment and control groups; MF, Mixed Findings showing significant benefits and harms; SF, Safe (primary outcome); NC, Not Collected or Analyzed yet; NR, Not Reported in publication yet listed as an outcome on clinicalTrials.gov. Route: IA, intraarterial; ICV, intracerebroventricular; IPU, intraputamenal; IV, intravenous; PO, peroral; NG, nasogastric intubation; SC, subcutaneous; TD, transdermal.
